# Overexpression of GDF15 protects kidneys from ischemia reperfusion injury and affects circular RNA expression

**DOI:** 10.3389/fcell.2025.1577625

**Published:** 2025-07-02

**Authors:** Cuilin Zhu, Qing Liu, Yale Su, Yixin Zhang, Aanal Patel, Adam Greasley, Jifu Jiang, Douglas Quan, Weiping Min, Kexiang Liu, Xiufen Zheng

**Affiliations:** ^1^ Department of Cardiovascular Surgery, The Second Hospital of Jilin University, Changchun, China; ^2^ Department of Pathology and Laboratory Medicine, Western University, London, ON, Canada; ^3^ Department of Cardiovascular Medicine, Ruijin Hospital, Shanghai Jiaotong University School of Medicine, Shanghai, China; ^4^ Department of Surgery, London Health Sciences Center, London, ON, Canada; ^5^ Department of Surgery, Western University, London, ON, Canada; ^6^ Department of MTOP, London Health Sciences Center Research Institute, London, ON, Canada; ^7^ Department of Oncology, Western University, London, ON, Canada; ^8^ Department of Microbiology and Immunology, Western University, London, ON, Canada

**Keywords:** GDF15, ischemia reperfusion injury, kidney, apoptosis, inflammation, circular RNA

## Abstract

**Background:**

Renal failure and dysfunction remain one of the most significant morbidities impacting patient’s life. Effective treatments still lack in the context of an increasing number of patients with renal failure. This study aims to investigate the impact of growth differentiation factor 15 (GDF15) in treating renal dysfunction and to explore its therapeutic potential.

**Methods:**

Renal injury was induced with a murine ischemia reperfusion injury (IRI) model. Mice overexpressing GDF15 (GDF15 transgenic (GDF15TG) mice, GDF15 knock out (GDF15 KO) mice and wild type (WT) mice all underwent IRI to test the effects of GDF15 on renal injury. Renal function and histopathological changes were measured 24 h after reperfusion. Cell apoptosis was detected by TUNEL and tissue inflammation was detected by myeloperoxidase (MPO) activity. qRT-PCR was conducted to determine the expression of genes and circular RNAs.

**Results:**

Overexpression of GDF15 reduced mortality of mice with lethal renal IRI whereas GDF15 deficiency increased the mortality. GDF15TG mice had better renal function with the lower levels of blood creatinine and blood urea nitrogen (BUN). Over-expression of GDF15 reduced kidney pathological changes, cell apoptosis, neutrophil infiltration and mortality. Over-expression of GDF15 also decreased the expression of apoptotic genes (high mobility group 1, HMGA1 and Bax), inflammatory genes IL-1β, IL-6, tumor necrosis factor (TNF-α), chemokine 1 (CK1), and senescent gene p21 whereas increases Bcl-XL, Importin 11 and CRIM1. IRI upregulated circular RNA Smad3 and reduced circular RNA Hipk3 and circular RNA Crim1, which was offset by GDF15.

**Conclusion:**

Over-expression of GDF15 protects renal function and prevents renal failure, highlighting its potential in treating renal failure.

## Introduction

Renal failure and dysfunction remain one of the most significant factor impacting patient’s life, which are associated with increased mortality, morbidity and prolonged hospitalization of patients (Smith et al., 2019; Kosieradzki and Rowinski, 2008). Renal ischemia reperfusion injury (IRI) remains one of the most signature causes, leading to renal failure and renal dysfunction. Renal IRI are complicated and currently characterized by cell apoptosis, mitochondrial oxidative stress, immune responses and a cascade of inflammatory processes (Smith et al., 2019; Gu et al., 2018; Chen et al., 2011; Zheng et al., 2006a; Zheng et al., 2006b). Despite various therapeutic strategies to reduce IRI, it is still a major problem in clinical practices and has no effective treatments. Thus, further investigation is required and new therapeutic targets need to be identified.

Growth differentiation factor 15 (GDF15), also known as macrophage inhibitory cytokine-1 (MIC-1), or nonsteroidal anti-inflammatory drug activated gene 1 (NAG-1), is a distinct member of the transforming growth factor β (TGF-β) superfamily. GDF15 is widely distributed in mammalian tissues and associated with apoptosis, inflammation, oxidative stress, hypoxia, telomere erosion, and cellular stress (Sharma et al., 2017; Zhang et al., 2017; Unsicker et al., 2013).

The role of GDF15 has been involved in different pathological kidney diseases. It has been reported that the GDF15 level is significantly correlated with increased risk of chronic kidney disease (CKD) progression and higher mortality risk in CKD patients with hemodialysis treatment (Nair et al., 2017). GDF15 has been reported to be predictive in patients with acute kidney injury (AKI) and is particularly helpful for risk stratification in AKI patients with normal levels of creatinine (Heringlake et al., 2016). We previously reported that over-expression of GDF15 can attenuate cardiac IRI and septic induced AKI (Zhang et al., 2017; Abulizi et al., 2017). However, the impact of GDF15 on renal IRI is not fully known.

Emerging evidence suggests that the pathophysiological mechanisms of IRI involve the interplay of inflammatory signaling cascades, programmed cell death pathways, and mitochondrial homeostasis regulation systems (Morciano et al., 2021). Recently emerging studies have shown that circular RNAs (circRNAs) which are generated by back-splicing and form circular structure with the 3′end of the RNA joining to the 5′end, have multiple functions including regulating gene expression, sponging miRNAs and interacting with RNA-binding proteins (Abdelmohsen et al., 2017; Du et al., 2017; Altesha et al., 2019). RNA sequencing has shown that circRNAs are highly expressed in kidney and circRNAs are differentially expressed in kidneys from AKI mice (Zhou et al., 2017). However, it is unknown whether GDG15 affects circRNAs during IRI.

This study aims to investigate the role of GDF15 in renal IRI and to demonstrate the involvement of circRNAs in renal IRI, as well as the effect of GDF15 on circRNAs, in order to better understand the molecular mechanism of IRI and to identify new targets for the development of new treatment for renal IRI.

## Materials and methods

### Animals

C57BL/6 wild type (WT) mice were purchased from Charles River Laboratories (Charles River Canada, Saint-Constant, Canada). GDF15 knock-out (KO) mice were kindly gifted by Professor Se-Jin Lee at John Hopkins University (Baltimore, MD, United States) (Hsiao et al., 2023). GDF15 Transgenic (TG) mice globally expressing human GDF15 were kindly gifted by Dr. Seung J. Baek at the University of Tennessee (Knoxville, TN, United States) (Baek et al., 2006). Heterozygous GDF15 TG/WT mice were bred between GDF15 TG mice and C57BL/6 WT mice, while GDF15 KO/WT mice were bred between GDF15 KO and C57BL/6 WT mice. All animal experiments in the study were approved by the Animal Care Use Committee of Western University.

### Mouse genotyping

DNA was extracted from the tail segment of mice and PCR was conducted for genotyping using a Red Extractin N-Amp TM Tissue PCR kit (Sigma), following the instruction of the manufacturer. Primer sequences were listed in Table 1. PCR products were separated in 1.5% agarose gels and visualized with Ethidium bromide.

**TABLE 1:** Primers for PCR.

Gene	Forward (5′-3′)	Reverse (5′-3′)
mGDF15	ACGAGCTACGGGGTCGC	CCCAATCTCACCTCTGGACTG
hGDF15	CTCCAGATTCCGAGAGTTGC	AGAGATACGCAGGTGCAGGT
BAX	AGGCCTCCTCTCCTACTTCG	AAATGCCTTTCCCCTTCCCC
BCL-XL	CCTCCTCCCCGACCTATGAT	CCCGGTTGCTCTGAGACATT
HMGA1	GCCCCAGCCCCATCTCAC	GGCGGTAACCCCATCTGTCTA
IL-6	CCGGAGAGGAGACTTCACAG	GGAAATTGGGGTAGGAAGGA
IL-1β	ACCTGGGCTGTCCTGATGAGAG	CCACGGGAAAGACACAGGTAGC
TNFα	CGTCAGCCGATTTGCTATCT	CGGACTCCGCAAAGTCTAAG
KC	CGCTCGCTTCTCTGTGCA	ATTTTCTGAACCAAGGGAGCT
P21	GGTGGTGGAGACCTGATGAT	CGGAACAGGTCGGACATCAC
IPO11	TGCAGGGCTCATCACCAACTTT	TTCCGCAGCACTTTTAACGATAGC
circSmad3	CCCCAGAGCAATATTCCAGA	CGTAATTCATGGTGGCTGTG
circCrim1	TGCCAGTGTATCAACGGAGA	GCAGTTCAGTTCCCCACACT
CircHipk3	CATGCTGACCTCAAACCAGA	ACACAACCGCTTGGCTCTAC
circFoxo3	GTGGGGAACTTCACTGGTGCTAAG	TTGTCCATGGAGACCGCACGCCG
CircAebp2	AGGGAAGGGACACAGTGTTG	CGACATGTATGGAGCGAATG
SMAD3	GCCTTTGTCTTCAGCACTCC	AGGCCAGCACAGGACTCTAA
CRIM1	TCCCAGTCCTCATCTTGCTGGCA	GTGTGCGACCCTGCGGGCTG
HIPK3	TGTACGTGTTGGGGCACTTA	CGGTTACAATGCAGCTGAGA
FOXO3	CAAGCTTTTGAGCCTGTGGG	TGCTTCGCCTACGCATAGTT
AEBP2	AGGGAAGGGACACAGTGTTG	CTGCGGCATTGTTCTGTAAA
GADPH	GGGGTGAGGCCGGTGCTGAGTAT	CATTGGGGTAGGAACACGGAAGG
Genotyping for WT mice	CCTGGAGACTGTGCAGGCAACTCTTG	GTGACACACCACTGTCTGTCCTGTGC
Genotyping for KO mice	GCTGTCCGGATACTCAGTCCAGAGG	CGCCTTCTTGACGAGTTCTTCTGAGGG

### Renal ischemia reperfusion injury model

Different strains of mice, aged 6–8 weeks, were anesthetized with an intraperitoneal injection of ketamine (100 mg/kg) and xylazine (20 mg/kg), and maintained under anesthesia with 1% isoflurane and oxygen mixture during surgery. Mice were placed on a heating pad to maintain a stable body temperature (37°C) during surgery. Following median abdominal incisions, the left renal artery and vein were carefully dissected, and a microvascular clamp (Roboz Surgical Instrument, Gaithersburg, MD) was placed on the left renal pedicle vessel for 55 min to induce reversible ischemic injury for kidney function testing or 60 min to induce lethal injury for mortality testing (Zager et al., 2014; Lerink et al., 2022). After ischemia, the clamp was removed and then the contralateral kidney was removed immediately. In this model, the renal function of the animals was completely dependent on IR injured kidneys. Thereafter, incisions were sutured, and the animals were allowed to recover with free access to food and water. The mice with 55 min-ischemia were sacrificed 24 h (hrs) postoperatively to collect blood and tissues for further experiments in order to assess renal function and injuries. The mice with 60 min-ischemia were monitored for 7 days post-operation, to observe mortality.

### BUN/creatinine measurement

Blood samples were obtained from the inferior vena cava or intra-cardiac 24 h after reperfusion. Serum creatinine levels and blood urea nitrogen (BUN) were measured by the core laboratory at the London Health Sciences Centre or at the lab of Matthew Mailing Centre for Translational Transplantation Studies, to assess renal functions.

### Histological detection

At 24 h post reperfusion, kidneys were harvested from mice, and tissue slices were fixed in 10% formalin, and then processed for histological examination using standard techniques. Formalin tissue was embedded in paraffin and 5 μm sections were stained with H&E, to examine the histological changes in the cortex and medulla. These sections were examined by a qualified pathologist in blinded fashion. The degree of injury was determined using a semi-quantitative graded scale: 0, no change; 1, less than 10%; 2, 11%–25%; 3, 26%–45%; 4, 46%–75% and 5, 76%–100%.

### Terminal deoxynucleotidyl transferase-mediated dUTP nick-end labelling (TUNEL) assay

Apoptosis in kidney tissues was detected by the TUNEL assay. Paraffin embedded tissues were sectioned and stained with an *in situ* cell death detection kit (Roche, Mississauga, ON, Canada) as described previously (Du et al., 2017). Apoptotic cells were counted under a microscope. The apoptosis score was determined using a five-point scoring system based on the apoptotic area involved as follows: 0, <10%; 1, 10%–25%; 2, 25%–50%; 3, 50%–75%; and 4, 75%–100%.

### Myeloperoxidase (MPO) staining

MPO staining was conducted to assess neutrophil infiltration in kidney tissue. Paraffin embedded tissue sections were stained with a polyclonal rabbit MPO antibody (1:100, NeoMarkers, Fremont, CA) and EnVision + anti-rabbit-HRP (Dako, Carpinteria, CA) to detect the activities of MPO as previously. The MPO score was determined using a five-point scoring system based on MPO-positive area involved as follows: 0, <10%; 1, 10%–25%; 2, 25%–50%; 3, 50%–75%; and 4, 75%–100%.

### RNA extraction

Total RNA was extracted using the RNeasy mini kit (Qiagen, Toronto, ON) according to manufacturer’s protocol. The concentration of RNA was determined by a NanoDrop UV-Vis Spectrophotometer (Fisher Scientific, Toronto, ON).

### qRT-PCR (quantitative reverse transcriptase-polymerase chain reaction)

cDNA was synthesized with 3 μg RNA using hexamer random primers (Invitrogen) and M-MulV reverse transcriptase enzyme (BioLabs Laboratories, Mississauga, Canada), following the manufacturer’s instructions. cDNA was diluted in 1:10 for qPCR.

Expression of HMGA1, BAX, Bcl-XL, IL-6, IL-1β, TNF-α, P21, KC, IPO11, GDF15, GADPH, FOXO3, SMAD3, CRIM1, HIPK3, AEBP2 and circular RNAs (circFoxo3, circSmad3, circCrim1, circHIPK3 and circAEBP2) were detected by qPCR. The primers used are listed in Table 1. SYBGreen mix (Wiscent, Mississauga, ON, Canada) was used and qPCR was conducted with 1x SYBGreen, 100 nmol/L forward/reverse primer and 1 µL diluted cDNA in a CFX Connection Real Time System (Bio-Rad laboratories), with the following protocol: 3 min at 94°C, followed by 40 cycles of 94°C for 30 s, 60^o^ for 20 s, 72°C for 30 s and 72°C for 10 min.

Expression of genes were quantitatively compared using the ΔΔCt method and GADPH was used as an internal loading control.

### Construction of the circRNA-miRNA-mRNA network

The circRNA-miRNA-mRNA regulatory network was constructed and visualized with online CircNet software.

### Statistical analysis

All the experiments were conducted independently at least 3 times (n ≥ 3). The statistical analyses were conducted using GraphPad Prism Version 10. Data were expressed by mean ± standard deviation. The t-test was used to compare the data between the two groups. One-way analysis of variance was used for comparisons among multiple groups. A p value <0.05 was considered significant. Kaplan-Meier analysis with the log-rank test was conducted to compare survival curves.

## Results

### Characterization of mouse genotype

**FIGURE 1:**
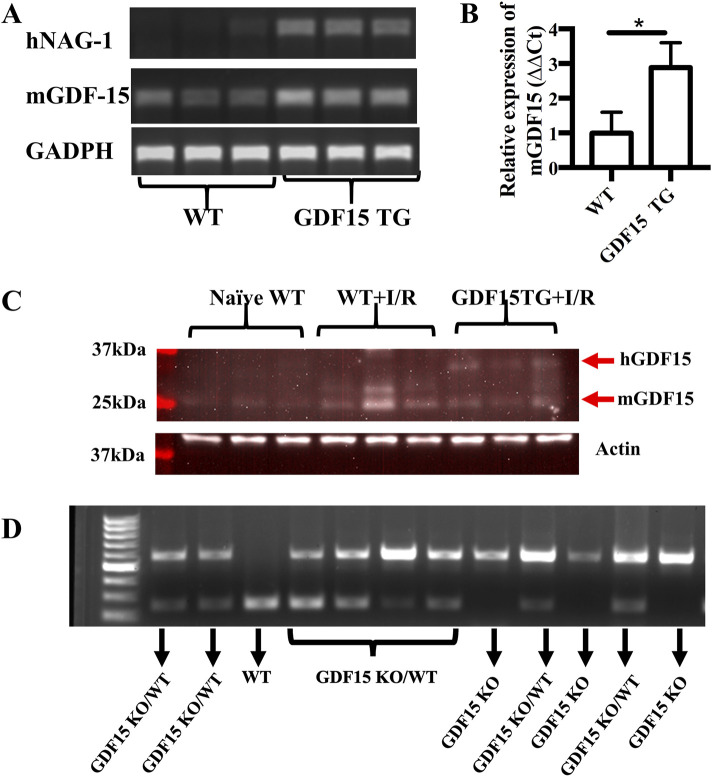
Characterization of mouse genotypes. Genotyping of mouse strain used in this study was performed. **(A,B)** Expression of human GDF15 (hGDF15) and mouse-derived GDF15 in GDF15TG mice and WT mice; **(C)** The expression of GDF15 proteins in mice underwent IRI. **(D)** Genotyping of GDF15KO and heterozygous GDF15KO/WT mice.

To better define the role of GDF15 in renal IRI, we used homozygous GDF15TG and GDF15KO mice as well as GDF15 heterozygous mice in this study. GDF15TG mice globally overexpressed human GDF15 or NAG-1 under the control of a chicken beta-actin promoter (CAG) (Baek et al., 2006), while GDF15KO mice whose entire mature C terminus sequence in the GDF15 gene was replaced by the neomycin resistance cassette (Hsiao et al., 2023). We first performed genotyping of our difference species to validate that GDF15TG mice not WT mice certainly highly expressed hGDF15 (Figure 1A). Mouse-derived GDF15 mRNA levels were also significantly higher (∼3 fold) in GDF15TG mice than that in WT mice under IR (Figure 1B). Western blot was performed to determine GDF15 at the protein level in ischemia conditions as shown in Figure 1C. Additional genotyping with PCR was performed for GDF15 TG/WT heterozygous mice, GDF15KO mice and WT mice. As shown in Figure 1D, WT mice had one band with size of 228bp, GDF15KO mice had one band with size of 598bp, while the GDF15 KO/WT heterozygous mice have the two bands (228bp and 598bp).

### Overexpression of GDF15 reduced mortality of mice with lethal renal IRI

**FIGURE 2:**
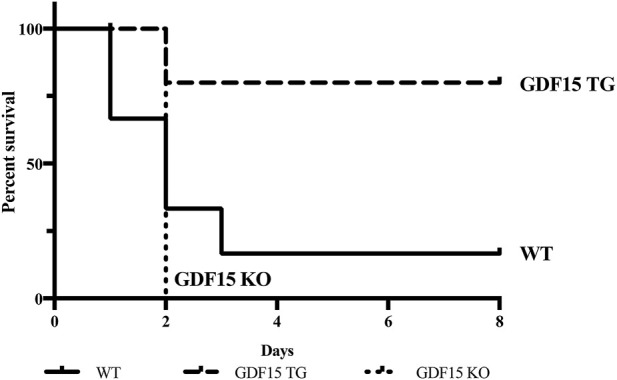
Deficiency of GDF15 increases mortality of mice with IRI. The left kidney was clamped for 60 min at 37°C to induce more severe IR injury. Animal survival was observed for 7 days post reperfusion, n ≥ 5, *p < 0.05. n = 6 for GDF15TG and WT mice and n = 5 for GDF15KO mice.

Firstly, we determined whether overexpression of GDF15 reduces IR-induced mortality. To induce lethal injury, kidneys of mice were clamped for 60 min at 37°C and the contralateral unclamped kidneys were removed immediately upon the beginning of reperfusion, as a result, the animal’s life depended on the function of the injured kidney. Six WT mice (n = 6) were observed: only one of which sustained to post operative day (POD) 7, while three died on POD1, one died on POD 2, and one died on POD3. Five out of six GDF15TG mice (n = 6) survived to POD7 and only one GDF15TG mouse died on POD2. In contrast, all GDF15KO mice (n = 5) died on POD2. The survival rates of animals for each group are presented in Figure 2, in which the GDF15TG mice had a significantly better survival rate and low mortality compared with WT (p < 0.05) and GDF15KO mice (p < 0.05). This demonstrated that over-expression of GDF15 reduced mortality, whereas GDF15 deficiency increased injury-related death.

### Overexpression of GDF15 is associated with improved renal function in mice with IRI

**FIGURE 3:**
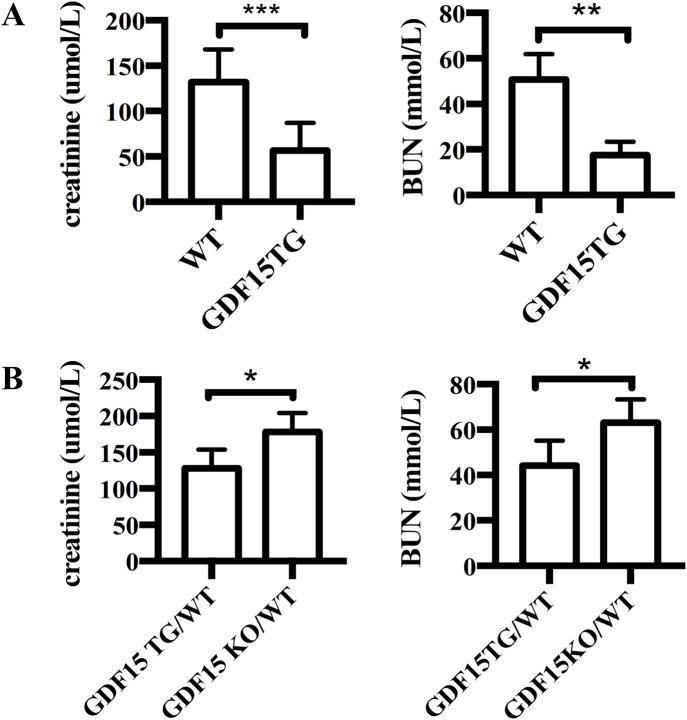
Overexpression of GDF15 protects renal function from IR injury. **(A)** Serum creatinine levels. The left kidney pedicle vessels were clamped for 55 min at 37°C, followed by 24hrs reperfusion. Renal function was determined by measuring the levels of serum creatinine **(A)** and BUN **(B)** 24 h after reperfusion. N = 6, *p < 0.05. ***p < 0.001.

To investigate the effect of GDF15 on renal IRI, GDF15TG mice (n = 6) and WT mice (n = 6) were used and a warm ischemia reperfusion (55 min ischemia at 37°C with 24 h reperfusion) model with less injury was applied. In this model, the left renal pedicle vessels (artery and vein) were clamped for 55 min at 37°C to induce warm ischemia, and the contralateral kidney was excised immediately after reperfusion. We found that GDF15TG mice behaved more actively in cages post operation than WT mice, indicating quicker recovery from surgeries. 24 h after reperfusion, the levels of creatinine and BUN in blood were measured to determine renal function. The results showed that the blood creatinine and BUN levels of GDF15TG mice were significantly lower compared with WT mice (p < 0.05) (Figure 3A), indicating that GDF15TG mice had better renal function than WT mice.

To further confirm the effect of GDF15 on IRI, we conducted the above experiments with heterozygous GDF15 TG/WT (n = 6) mice and GDF15 KO/WT (n = 6) mice. As shown in Figure 3B, the creatinine levels in GDF15 TG/WT mice were 128 ± 10.48 μmol/L and 178.4 ± 11.45 μmol/L in GDF15 KO/WT, respectively (p < 0.05), while the BUN levels were 44.08 ± 4.519 μmol/L in GDF15 TG/WT and 63.08 ± 4.156 μmol/L in GDF15 KO/WT, respectively (p < 0.05), indicating that loss of GDF15 results in worse renal function.

### Overexpression of GDF15 reduced tissue injury and cell apoptosis

**FIGURE 4:**
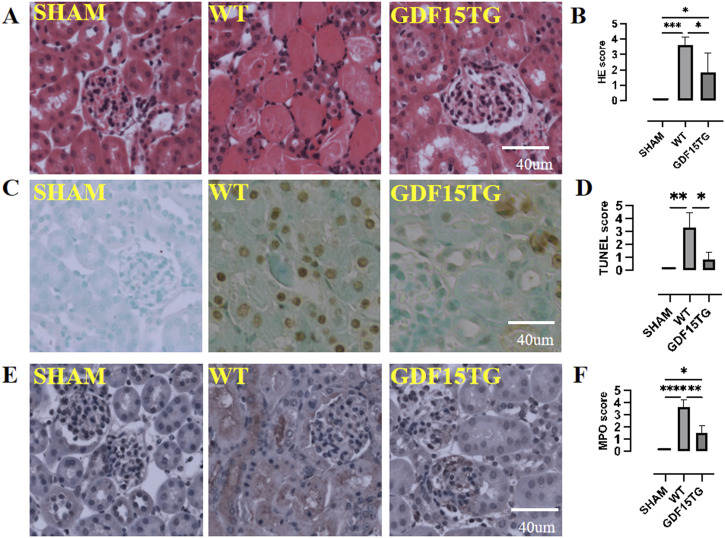
Overexpression of GDF15 reduces histopathological changes, cell apoptosis, and neutrophil infiltration. Animal were collected for histopathological changes by HE staining, cell apoptosis by TUNEL assays, and neutrophil infiltration by MPO staining 24hrs after reperfusion. Kidney injury, apoptosis, and MPO was semi-quantified using a five point score system based on degeneration of kidney: 0: no injury or no apoptosis, or MPO staining positive; 1: <10% area; 2: 11%–30% area; 3: 31%–60%; 4: 61%–90%; 5: >91%. **(A)** Representative images of HE staining; **(B)**. Kidney injury score. **(C)** Representative images of TUNEL; **(D)** TUNEL score; **(E)** Representative images of MPO; **(F)** MPO score. n = 3-6, *p < 0.05.

Histopathological changes of kidneys were examined 24 h after reperfusion to assess IRI. As shown in Figure 4A, WT mice with 55 min ischemia suffered from severe injury and cell death, with loss of normal structure in the tubule and glomerulus. In contrast, the GDF15TG mice had better kidney structures, and their main pathological changes were manifested in cell swelling and degeneration, instead of cell death. The injury level of GDF15TG kidneys was significantly lower compared with WT mice (Figure 4B).

Consistent with renal function, the detrimental histopathological changes in kidneys from heterozygous GDF15 TG/WT mice were significantly less severe compared with GDF15 KO/WT mice and WT mice (data now shown).

Apoptosis *in situ* was detected by TUNEL assays. As depicted in Figures 4C,D, IR induced kidney cell apoptosis. However, GDF15TG mice had significantly fewer TUNEL-positive apoptotic cells than WT mice.

Neutrophil infiltration occurs at an early period after IR leading to inflammation and injury. MPO activity was examined to evaluate neutrophil infiltration in kidney tissues. As shown in Figures 4E,F, there were fewer MPO-positive cells in the kidneys from GDF15TG mice compared with that from WT mice, indicating that over-expression of GDF15 reduced neutrophil infiltration.

### Overexpression of GDF15 reduced the expression of pro-apoptotic and pro-inflammatory genes

In order to understand the molecular mechanism, we detected expression of genes associated with cell apoptosis and inflammation.

**FIGURE 5:**
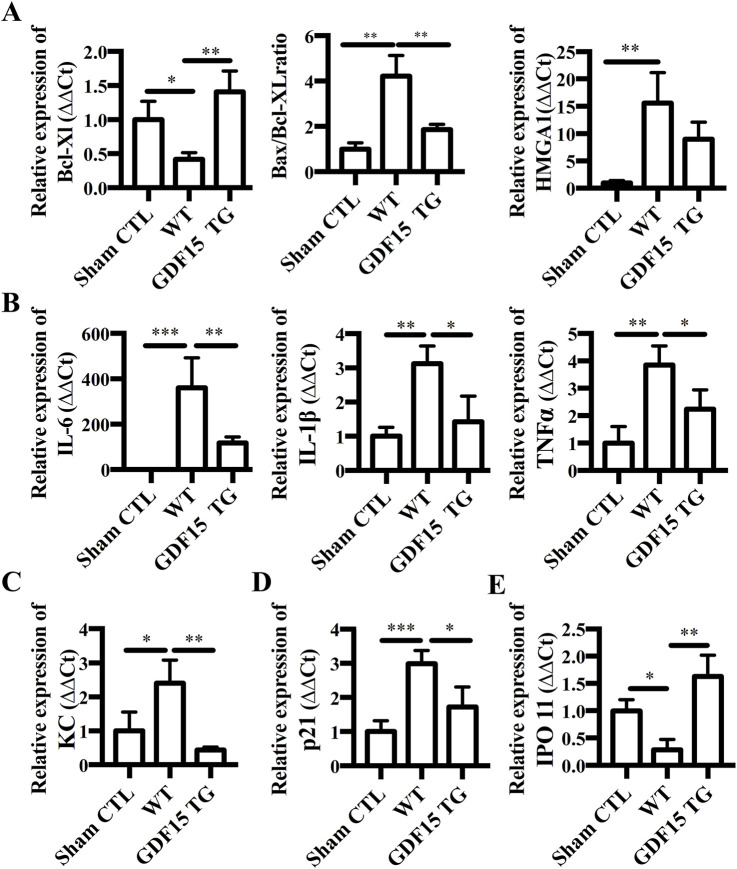
Overexpression of GDF15 reduces expression of apoptotic, inflammatory genes, KC, P21 and IPO 11. Total RNA was extracted from kidneys 24 h after IR and gene expression was measured by qRT-PCR. Relative gene expression was expressed as ΔΔCt, where GAPDH was used as an internal control. **(A)** Expression of Bcl-XL, Bax and HMGB1. **(B)** Expression of IL-6, IL-1β, and TNFα. **(C)** KC; **(D)** p21; **(E)** IPO 11. n = 4 per group, *p < 0.05.

Shown in Figure 5A, the expression of anti-apoptotic gene Bcl-xl was significantly decreased in IR injured kidneys from WT mice compared with the sham surgical control (p = 0.0570), while GDF15TG mice expressed significantly higher levels of Bcl-xl compared with WT mice. The ratio of Bax/Bcl-xl, which is often used to assess apoptosis, was also significantly increased in WT mice with IRI compared to the sham control (p < 0.05). However, the ratio of Bax/Bcl-xl was significantly lower in GDF15TG mice than in WT mice, demonstrating anti-apoptotic function of GDF15 in renal IRI. We also found that the expression level of high mobility group protein A1 (HMGA1), which is a gene used to measure cell death, was decreased in GDF15TG mice, compared with WT mice (Figure 5A), indicating the anti-cell death function of GDF15.

Inflammatory response plays an important role in regulating IRI and GDF15 has been reported to have an anti-inflammatory function (Breit et al., 2011). Accordingly, we detected the expression of inflammatory cytokine genes IL-6, IL-1β and TNFα. As shown in Figure 5B, the expression of IL-6, IL-1β, and TNFα was significantly increased in WT mice with renal IRI compared with the sham surgery group (p < 0.05), while the increased expression of IL-6, IL-1β and TNFα was significantly offset in GDF15TG mice (p < 0.05).

Expression of CXC chemokine keratinocyte‐derived chemokine (KC, also known as CXCL1) was also detected. We found that IR increased the expression levels of KC compared with sham surgery (p < 0.05) whereas over-expression of GDF15 significantly mitigated the increase in KC expression (p < 0.05) (Figure 5C).

P21, also known as cyclin dependent kinase inhibitor 1A (CDKN1A), is a critical cell cycle regulatory protein that promotes cell cycle arrest by inhibiting multiple cyclin-dependent kinases and has been reported to be upregulated by diverse forms of AKI. We found that the expression of P21 mRNA was significantly increased in the WT group compared with the sham surgery group (p < 0.05), while it was significantly decreased in the GDF15TG group compared with the WT group (Figure 5D).

Additionally, the expression of Importin 11 (IPO 11) which functions as a nuclear transport receptor was significantly reduced in WT mice compared with sham surgery (p < 0.05), while in GDF15TG mice the expression level of IPO was significantly higher than WT group (p < 0.05) (Figure 5E).

### GDF15 regulated circRNA expression in renal IRI

**FIGURE 6:**
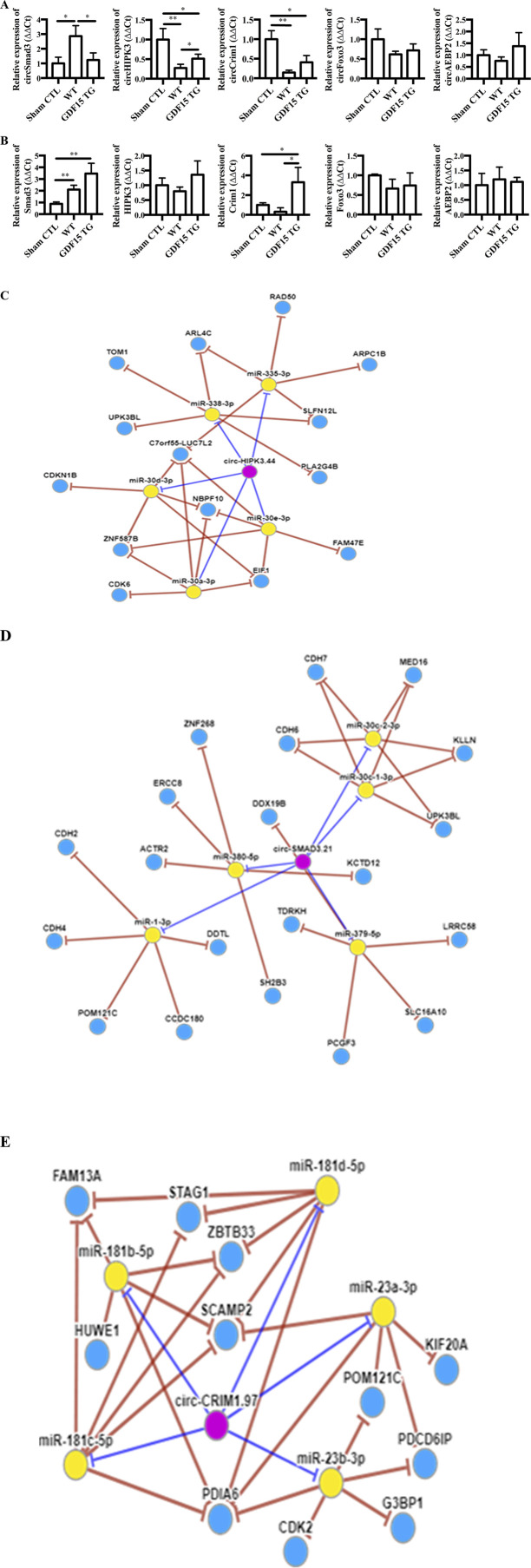
GDF15 alters the expression of circRNAs and their home genes. Total RNA was extracted from kidneys 24 h after I/R injury. The expression of circular RNAs (circSmad3, circHipk3, circCim1, circAebp2 and circFoxo3) **(A)** and their home genes **(B)** was detected by qRT-PCR. n = 3 or 4. GAPDH was used as a loading control, *p < 0.05. **(C–E)** CircRNA-miRNA-gene network. A circRNA-miRNA-gene network was constructed using circNet program (http://syslab5.nchu.edu.tw/CircNet). **(C)** circHipk3; **(D)** circSmad 3; **(E)** circCrim1. n = 4 per group, *p < 0.05.

circRNAs are a new class of non-coding RNA and growing evidence shows that they are involved in different pathologies. The expression of circRNA Smad3 (circSmad3), circRNA Hipk3 (circHipk3), circRNA Crim1 (circCrim1), circRNA Foxo3 (circFoxo3) and circRNA Aebp2 (circAebp2), which we found changed in IR injured heart cells, was determined by qRT-PCR in kidneys. Compared with sham surgery, IR significantly increased the expression of circSmad3, but decreased the expression of circHipk3 and circCrim 1, indicating the involvement of circRNAs in renal IRI. However, the alterations of these three circRNA were attenuated in GDF15TG mice (Figure 6A). There were no significant differences in the expression of circFoxo3 and circAebp2 among the groups.

The mRNA levels of SMAD3, FOXO3, AEBP2, HIPK3, and CRIM1 genes in which the above five circRNAs are spliced were also measured, respectively. Our results showed that kidney tissues generally expressed reasonable levels of SMAD3, FOXO3, AEBP2, HIPK3, and CRIM1 mRNA (Figure 6B). There were no significant changes in the expression of FOXO3, HIPK3 and AEBP2 mRNA among the three groups. In contrast, SMAD3 was upregulated by IR whereas CRIM1 was decreased, compared to the sham control. Interestingly, GDF15 over-expression increased the expression of SMAD3 and CRIM1 compared with WT mice, where only the increases of CRIM1 were statistically significant.

Additionally, circRNA-miRNA-gene Regulatory Network was constructed using circNet program (Figures 6C–E), showing the top five miRNAs each circRNA might interact with and the possible targets of those miRNAs.

## Discussion

In this study, we demonstrate that overexpression of GDF15 protects mice from renal IRI shown by improving renal functions, decreasing histopathological changes, decreasing cell apoptosis/death and neutrophil infiltration, reducing the expression of pro-apoptotic genes, pro-inflammatory genes and cell senescence related gene. Over-expression of GDF15 also reduces mortality of mice with lethal renal IRI. CircRNAs are involved in renal IRI and GDF15 affects the expression of circCrim1, circSmad3, and circHipk3. Our data, for the first time, demonstrates that the overexpression of GDF15 provides a multitude of protective benefits to IR injured kidneys and might provide a new therapeutic target for preventing renal IRI.

Our body is empowered with a self-adoptive defense system against stress or an insult through controlling gene expression and signaling pathway activation. GDF15 is an immediate reaction gene under stress and is quickly upregulated in response to stress and its over-expression aims to prevent cells and organs from further damage in cardiac IR models (Kempf et al., 2006; Karan et al., 2009; Kempf et al., 2011). Dokuyucu et al. reported that IR increased the expression of GDF15 in a rat renal IR model with 60 min of ischemia plus 48 h reperfusion, but the increase was not statistically significant (D et al., 2014). Our data here show that deficiency of GDF15 exacerbated renal IRI and increased mortality of mice. In contrast, over-expression of GDF15 protects kidneys from IRI. The protective effects of GDF15 overexpression were approved to be valid in both slight renal injury (55 min of ischemia model) and severe renal injury causing lethal death (60 min of ischemia model) (Zager et al., 2014; Lerink et al., 2022). Similar to our previous study of heart transplantation (Zhang et al., 2017), over-expression of GDF15 reduces cell apoptosis and inflammation, and the expression of apoptotic genes and pro-inflammatory genes.

We also demonstrated the effect of GDF15 on chemokine expression. KC, one of the CXC chemokines, mainly attracts and activates polymorphonuclear leukocytes including neutrophils to sites of acute inflammation and is upregulated in kidneys with IRI (Stroo et al., 2010). Neutralizing KC protects renal function against IRI (Kempf et al., 2011). In this study, we observed that IR upregulated KC expression in kidneys and attracted more neutrophils infiltrating into the kidneys. The elevations of KC mRNA and neutrophil infiltration in the kidneys from GDF15TG mice were significantly attenuated compared to WT mice, indicating GDF15 limiting neutrophil infiltration via reducing expression of KC. Despite it has not been reported that GDF15 regulates KC in kidneys, hearts or other solid organs, a cancer study showed that an inhibition of GDF15 increased the plasma levels of KC (D et al., 2014), which indirectly supports our result.

It has been reported that GDF15 inhibits transforming growth factor β-activated kinase 1 (TAK1)/NF-κB activation, limiting neutrophil migration (Zhang et al., 2023). Our results reported here show that over-expression of GDF15 reduced the expression of pre-inflammatory cytokines IL-1β and IL-6 which are transcribed by the NF-KB pathway, indirectly suggesting that GDF15 overexpression attenuates inflammation and limits neutrophil infiltration into the kidneys via inactivation of the NF-KB pathway. Additionally, GDF15 has been reported to inhibit the activation of the small GTPase Rap1 and to block activation of β2-integrin affinity and clustering, thereby preventing neutrophil adhesion and migration in a psoriasis models and a cardiac infarction model (Zhang et al., 2023; Zhang et al., 2016). It is also possible that GDF15 overexpression limits neutrophil infiltration into IR injured kidneys through regulating GTPase Rap1 and β2-integrin affinity, which needs to be validated in a future study.

IR has been reported to induce cell senescence in kidneys (Stasi et al., 2025). GDF15 has been considered as a potential and high-priority candidate against aging and age-related disorders, which is an important characteristic of cell senescence phenotype. Literature has shown that long ischemic time (30 and 45 min), not short ischemic time, upregulated senescence molecule p21 in the tubular cells of kidneys. Targeting p21 with the selective p53 inhibitor pifithrin-alpha before ischemia could prevent acute renal failure (Nishioka et al., 2014). In the current study, we found that 55 min of IR increased p21 in the kidneys. By contrast, over-expression of GDF15 offsets the increase of p21. Moreover, we found that IR decreased the expression of importin 11 (IPO 11) but reversed by GDF15 over-expression. IPO 11 is a nuclear transport receptor directly interacting with Ran GTPase and UbcM and transport UbcM2 (Plafker and Macara, 2000). It has been reported that IPO 11 is associated with apoptosis and its downregulation increased the expression of apoptotic genes and cell apoptosis (Ni et al., 2021). Although there are no reports about IPO 11 expression and function in kidneys under IR, our results show that an involvement of IPO 11 and its association with GDF15. Our data suggest GDF15 protecting kidneys from IRI through regulating p21 and IPO11 and the possible roles of p21 and IPO11 in renal IRI, despite further investigations are needed to elucidate the functions of GDF15 in regulating cell senescence and component nucleocytoplasmic transport in kidney IRI and to dissect the potential pathways involved.

CircRNAs are a new type of non-coding RNAs that regulate gene expression and play vital roles in physiological and pathological processes (Altesha et al., 2019; Jin et al., 2020; Wang et al., 2021; Li et al., 2021; Li and Xing, 2024). RNA sequencing showed that circRNAs are abundant in the kidney and that AKI caused by 30 min ischemia and 24hrs reperfusion significantly alters expression of a number of circRNAs (Zhou et al., 2017). Our data also confirmed that circRNAs are expressed in the kidneys. We further demonstrated that IR changes the expression of circSmad3, circCrim1 and circHipk3, but two other circRNAs (circFoxo3 and circAebp2) were not changed. IR upregulates the expression of circSmad3, while decreasing circCrim1 and circHipk3. These three circRNAs were not changed in the AKI model with 30min ischemia reported by literature (Zhou et al., 2017). A more recent study reported that circHipk3 was upregulated in kidney in a mouse IRI model in which both renal pedicles were clamped for 25 min followed by 6 h reperfusion (Zhengbiao et al., 2023). The different profiles of circRNA expression among different models and tissues suggest the model - and tissue-specificity of circRNAs, which has previously been decomented (Altesha et al., 2019; Xia et al., 2017). Moreover, our study shows that over-expression of GDF15 decreases the expression of circSmad3 but increases circCrim1 and circHipk3, suggesting that circSmad3 is caustic circRNA while circCrim1 and circHipk3 are protective in renal IRI. As a member of the TGF-β family, GDF15 increased SMAD3 and could regulate TGF β/SMAD3 signaling, which may regulate circRNA biogenesis. Since SMAD3 mRNA was increased by GDF15, it is possible that GDF15 modulates circSmad3 via regulating its splicing, which needs to be demonstrated in future. The network of circRNA and miRNA listed the top five miRNAs which might be targeted by circSmad3, circCrim1 and circHipk3, respectively. Some of them, for example, miR-30,^41^ miR-1,^42^ miR-181, miR-23,^43^ and miR-383,^44^ have been associated with IRI. There is little information about circSmad3 and circCrim1 in literature, whereas circHipk3 has been reported to regulate cell growth (Kai et al., 2018), proliferation (Che et al., 2019; Cai et al., 2019), autophagy (Chen et al., 2019), and cell apoptosis (Zhengbiao et al., 2023), through interaction with miRNAs. The circRNA-miRNA-mRNA interaction could be validated by luciferase reporter assays. Nonetheless, none of the above circRNAs have been reported on renal IRI and their functions need to be further investigated. Additionally, this study also demonstrated that IR reduced the expression of CRIM1 mRNA, which was reversed by over-expression of GDF15, implying that CRIM1 might be a new target for IRI.

Accumulating preclinical evidence has elucidated the therapeutic promise of GDF15 in various diseases. Administration of murine mature GDF15 have been proven to show therapeutic effects against metabolic disorders (Tsai et al., 2018). Mice that express GDF15 expression have reduced nonalcoholic steatohepatitis, an inflammatory condition in the liver (Kim et al., 2018). These mechanistic insights establish a robust molecular foundation for developing GDF15-targeted therapies, with particular translational potential in metabolic syndrome-related pathologies and other diseases.

There are several limitations of our study. Firstly, the current study used a severe AKI model where the contralateral kidney was immediately removed after I/R. A less severe AKI model in which the contralateral kidney remains intact for an extended period will help validate the role of GDF15 in promoting kidney tissue repair, which needs to be used in future. Secondly, we used GDF15KO and GDF15TG mice in which GDF15 was globally knocked out and overexpressed. GDF15 effect is systemic and global knockout of GDF15 may affect other organs. Cardiac complications may also contribute to the increased mortality in GDF15KO mice with IRI, in addition to renal dysfunction. Kidney-specific GDF15 modulation is needed to conduct in future studies. Thirdly, measurement of urinary GDF15 would also be beneficial in further understanding the role of GDF15 in regulating IRI. Fourthly, more experiments elaborating the mechanisms of GDF15 regulating circRNA and the interaction of circRNA-miRNA-mRNA need to be conducted.

In conclusion, increasing expression of GDF15 in renal tissues is beneficial and over-expression of GDF15 protects kidneys from IRI through inhibition of cell apoptosis, inflammation and senescence. We demonstrated that circRNA is associated with renal IRI and GDF15 overexpression affects the expression of circSmad3, circHipk3 and circCrim1 in IR injured kidneys. Thus, GDF15/circRNA might be a promising therapeutic target for preventing ischemic damage to kidneys. With preclinical study performed, more clinical trials warrant investigation.

## Data Availability

All data in this study are available upon reasonable request.
